# Plasma Pharmacokinetics of High-Dose Oral versus Intravenous Rifampicin in Patients with Tuberculous Meningitis: a Randomized Controlled Trial

**DOI:** 10.1128/AAC.00140-21

**Published:** 2021-07-16

**Authors:** Sean Wasserman, Angharad Davis, Cari Stek, Maxwell Chirehwa, Stephani Botha, Remy Daroowala, Marise Bremer, Mpumi Maxebengula, Sonya Koekemoer, Rene Goliath, Amanda Jackson, Thomas Crede, Jonathan Naude, Patryk Szymanski, Yakoob Vallie, Muhammed S. Moosa, Lubbe Wiesner, John Black, Graeme Meintjes, Gary Maartens, Robert J. Wilkinson

**Affiliations:** a Wellcome Centre for Infectious Diseases Research in Africa, Institute for Infectious Disease and Molecular Medicine, University of Cape Town, Cape Town, South Africa; b Division of Infectious Diseases and HIV Medicine, Department of Medicine, University of Cape Town, Cape Town, South Africa; c Francis Crick Institute, London, United Kingdom; d Faculty of Life Sciences, University College London, London, United Kingdom; e Department of Infectious Diseases, Imperial College, London, United Kingdom; f Department of Medicine, University of Cape Town, Cape Town, South Africa; g Division of Clinical Pharmacology, Department of Medicine, University of Cape Town, Cape Town, South Africa; h Livingstone Hospital Complex, Eastern Cape Department of Health, Port Elizabeth, South Africa; i Mitchells Plain Hospital, Western Cape Department of Health, Cape Town, South Africa; j New Somerset Hospital, Western Cape Department of Health, Cape Town, South Africa

**Keywords:** human immunodeficiency virus, pharmacokinetics, rifampicin, tuberculous meningitis

## Abstract

Higher doses of intravenous rifampicin may improve outcomes in tuberculous meningitis but are impractical in high-burden settings. We hypothesized that plasma rifampicin exposures would be similar between oral dosing of 35 mg/kg of body weight and intravenous dosing of 20 mg/kg, which has been proposed for efficacy trials in tuberculous meningitis. We performed a randomized parallel-group pharmacokinetic study nested within a clinical trial of intensified antimicrobial therapy for tuberculous meningitis. HIV-positive participants with tuberculous meningitis were recruited from South African hospitals and randomized to one of three rifampicin dosing groups: standard (oral 10 mg/kg), high dose (oral 35 mg/kg), and intravenous (20 mg/kg). Intensive pharmacokinetic sampling was done on day 3. Data were described using noncompartmental analysis, and exposures were compared by geometric mean ratios (GMRs). Forty-six participants underwent pharmacokinetic sampling (standard dose, *n* = 17; high-dose oral, *n* = 15; intravenous, *n* = 14). The median CD4 count was 130 cells/mm^3^ (interquartile range [IQR], 66 to 253 cells/mm^3^). The rifampicin geometric mean area under the concentration-time curve from 0 to 24 h (AUC_0–24_) values were 42.9 μg · h/ml (95% confidence interval [CI], 24.5 to 75.0 μg · h/ml) for the standard dose, 295.2 μg · h/ml (95% CI, 189.9 to 458.8 μg · h/ml) for the high oral dose, and 206.5 μg · h/ml (95% CI, 154.6 to 275.8 μg · h/ml) for intravenous administration. The rifampicin AUC_0–24_ GMR was 1.44 (90% CI, 0.84 to 2.21) and the maximal concentration of drug in serum (*C*_max_) GMR was 0.89 (90% CI, 0.63 to 1.23) for high-dose oral administration with respect to intravenous dosing. The plasma rifampicin AUC_0–24_ was higher after an oral 35-mg/kg dose than with intravenous administration at a 20-mg/kg dose over the first few days of tuberculosis (TB) treatment. The findings support oral rifampicin dosing in future tuberculous meningitis trials.

## TEXT

Tuberculous meningitis (TBM) in HIV-positive people carries a mortality rate approaching 60% (1, 2), and despite antituberculosis (anti-TB) therapy, half of all survivors suffer significant neurological sequelae (3). One strategy to potentially improve outcomes is enhanced bacterial killing through optimized antibiotic therapy (4).

Rifampicin is the key agent in TBM therapy; its exclusion from treatment worsens outcomes, and there is a high mortality rate from rifampicin-resistant TBM (5). However, rifampicin is highly protein bound (6), and the cerebrospinal fluid (CSF) penetration of total drug is poor (7), rarely exceeding the MIC of Mycobacterium tuberculosis (8–10). Studies of pulmonary TB have shown that the bactericidal activity is related to the rifampicin area under the concentration-time curve (AUC) (11, 12) and that microbiological outcomes are improved at higher doses, up to 40 mg/kg of body weight (13–15). A small randomized controlled trial showed a survival benefit with the use of intravenous (i.v.) rifampicin at 13 mg/kg for Indonesian adults with TBM (16), which had plasma exposures similar to those with oral rifampicin at 20 mg/kg (17). A modestly increased oral rifampicin dose of 15 mg/kg did not improve survival in a phase 3 trial (2); however, higher doses may be required to improve outcomes. A meta-analysis of Indonesian TBM trials demonstrated a rifampicin exposure-response effect for survival in TBM but with poor precision (18).

Several clinical trials (ClinicalTrials.gov identifier NCT04145258, ISRCTN identifier ISRCTN42218549, and ClinicalTrials.gov identifier NCT03537495) are currently investigating the safety and efficacy of oral rifampicin doses of up to 35 mg/kg for TBM. Because rifampicin has dose-dependent bioavailability (19) and exhibits nonlinear increases in exposure with higher doses (12, 20, 21), 35 mg/kg orally may attain or even exceed intravenous plasma exposures at doses higher than 13 mg/kg. Existing population pharmacokinetic (PK) models can predict plasma rifampicin concentrations at doses of up to 40 mg/kg orally (19), but this has not been done for intravenous administration, where exposure is unaffected by the prehepatic first-pass effect (19). This knowledge gap has important implications for TBM trials and the ultimate deployment of intensified antimicrobial therapy for TBM in resource-limited settings as intravenous rifampicin has limited availability, and its use will be associated with increased costs, hospitalizations, and complications relating to peripheral venous catheterization.

Based on existing PK models of rifampicin (19, 20) and data showing equivalent AUCs between 13 mg/kg given intravenously and 20 mg/kg given orally (17), we hypothesized that plasma rifampicin exposures will be similar between 35 mg/kg given orally and 20 mg/kg given intravenously, which has been proposed for efficacy trials in TBM. To test this, we performed a randomized parallel-group PK study nested within a clinical trial of high-dose rifampicin for HIV-associated TBM.

## RESULTS

### Participants.

**FIG 1:**
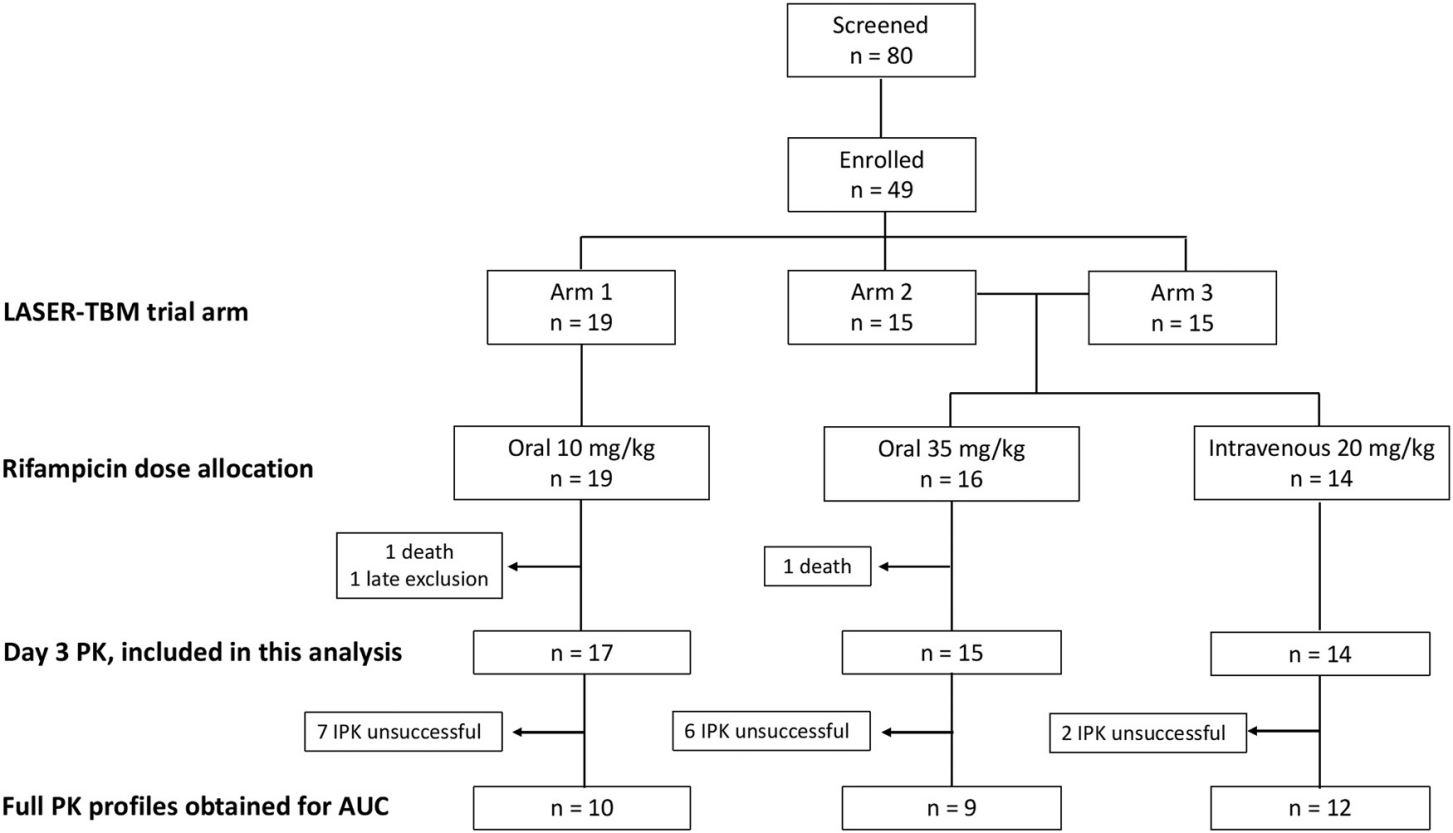
Trial consort flow diagram. Arm 1, standard TB therapy; arm 2, high-dose rifampicin plus linezolid; arm 3, high-dose rifampicin plus linezolid and aspirin. IPK, intensive PK; AUC, area under the concentration-time curve up to 24 h. Adequate PK profiles are those with at least two observations in the elimination phase.

Forty-nine participants were enrolled in the parent trial, but 2 participants died, and 1 was withdrawn due to late exclusion (estimated glomerular filtration rate [eGFR] of >20 ml/min) prior to receiving the investigational product: 46 participants underwent intensive PK sampling and were included in this analysis (Fig. 1).

Baseline characteristics were well balanced across rifampicin dosing groups (Table 1). One-third of participants had definite TBM, with the majority (61%) having British Medical Research Council (MRC) grade 1 disease. The median duration of antituberculosis therapy before the PK visit was 5 days (interquartile range [IQR], 4 to 6 days) and was similar across arms (although the PK visit occurred on study day 2 or 3, up to 5 days of standard TB treatment were allowed prior to enrollment). Rifampicin was crushed and administered by syringe for 6 participants (2 in the high-dose group and 4 in the standard-dose group). The duration of intravenous infusion was 60 min for all participants except two (15 min and 68 min).

**TABLE 1:** Baseline characteristics^*a*^

Parameter	Value for group			*P* value
	Oral 10 mg/kg (*n* = 17)	Oral 35 mg/kg (*n* = 15)	i.v. 20 mg/kg (*n* = 14)	
Median age (yrs) (IQR)	38 (34–47)	41 (36–45)	37 (30–43)	0.26

% (no.) of female participants	47 (8)	33 (5)	50 (7)	0.62

% (no.) of participants of ethnicity^*b*^				0.26
African	82 (14)	80 (12)	93 (13)	
Caucasian	12 (2)	0	0	
Mixed race	6 (1)	20 (3)	7 (1)	

Median wt (kg) (IQR)	64 (54–77)	60 (53–80)	59 (54–62)	0.67

Median BMI (kg/m^2^) (IQR)	25 (22–32)	22 (20–23)	22 (19–23)	0.08

Median CD4 count (cells/μl) (IQR)	130 (64–253)	131 (45–204)	145 (96–333)	0.43

% (no.) of patients with ART status				0.42
On ART	29 (5)	27 (4)	36 (5)	
ART naive	53 (9)	27 (4)	36 (5)	
Previous ART	18 (3)	47 (7)	29 (4)	

% (no.) of patients with TBM diagnosis				0.65
Definite TBM	41 (7)	27 (4)	29 (4)	
Possible TBM	29 (5)	53 (8)	36 (5)	
Probable TBM	29 (5)	20 (3)	36 (5)	

% (no.) of patients with MRC grade				0.59
Grade 1	59 (10)	53 (8)	71 (10)	
Grade 2	41 (7)	47 (7)	29 (4)	
Grade 3	0	0	0	

Modified Rankin score (IQR)	3 (1–5)	3 (1–5)	3 (1–4)	0.95

Median duration of TB treatment before PK visit (days) (IQR)^*c*^	5 (4–6)	5 (3–6)	6 (4–7)	0.65

Median total rifampicin dose (mg) (IQR)	600 (450–750)	2,100 (1,800–2,700)	1,350 (1,200–1,350)	<0.001

Median rifampicin dose (mg/kg) (IQR)	9 (8–10)	34 (33–36)	22 (22–24)	<0.001

a ART, antiretroviral therapy; BMI, body mass index; MRC, British Medical Research Council.

b Self-reported.

c Participants were allowed to receive up to 5 days of TB treatment prior to trial enrollment.

### PK data.

There was a total of 304 PK observations, 40 of which were below the limit of quantification (BLQ). There were 35 PK profiles with at least two observations in the elimination phase available for AUC from 0 to 24 h (AUC_0–24_) analysis after imputation: 12 in the standard-dose group, 10 in the high-dose oral group, and 13 in the i.v. group. Trough concentrations were imputed for 9 participants due to missing 24-h concentrations in 8 and dosing prior to the 24-h concentration in 1. The predose concentration was imputed for a single participant because of late dosing the day before the PK visit.

**FIG 2:**
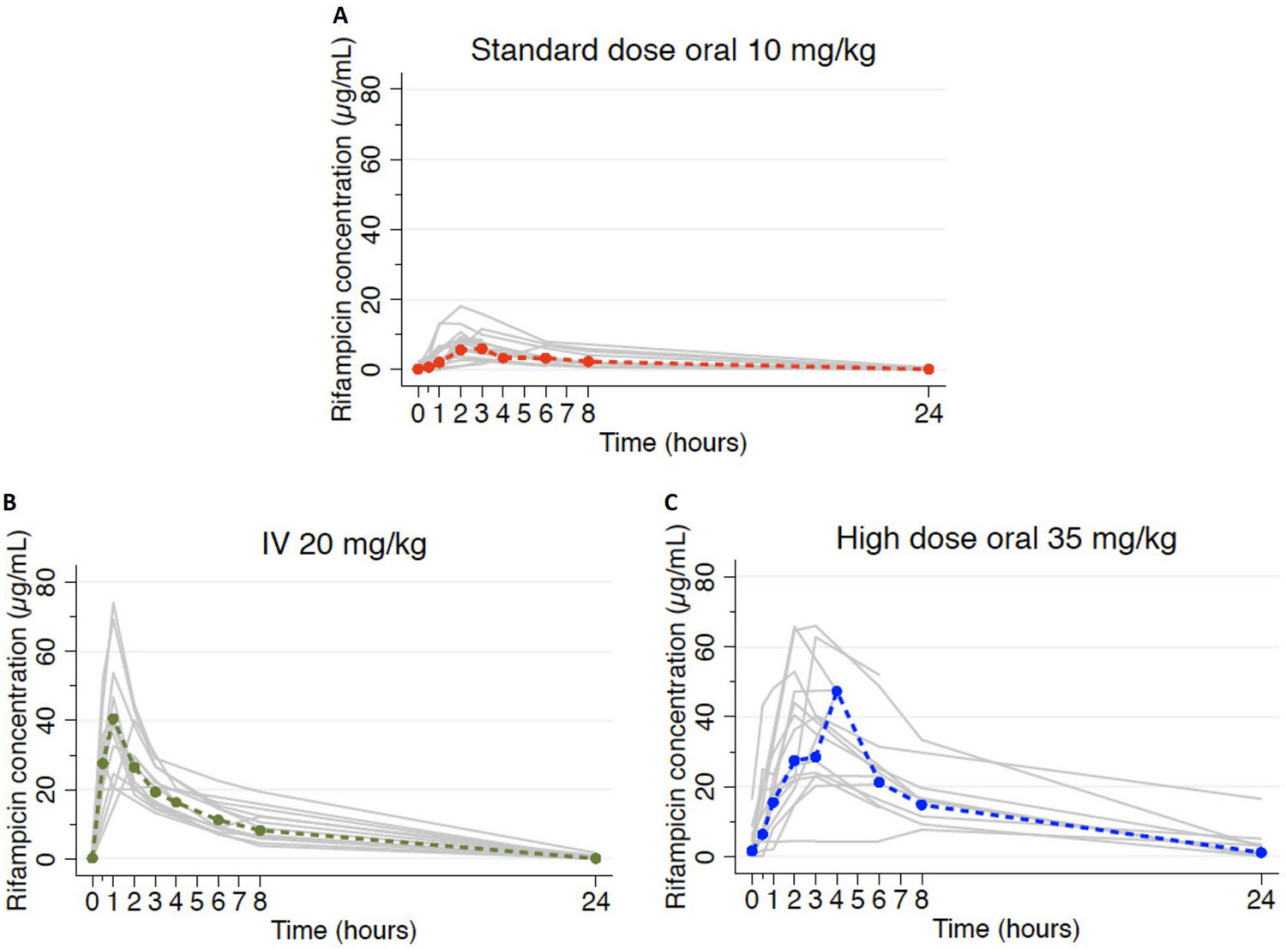
Individual concentration-time profiles. PK profiles for all participants by rifampicin dose allocation are shown. Gray lines indicate individual profiles, and colored dashed lines indicate geometric means. IV, intravenous.

Concentration-time profiles in Fig. 2 demonstrate much higher concentrations in the high-dose and i.v. groups than with standard dosing. There was high interindividual variability in plasma concentrations, particularly in the oral dosing groups (standard-dose maximal concentration of drug in serum [*C*_max_] percent coefficient of variance [%CV], 52; high-dose oral %CV, 48; i.v. %CV, 38), which also showed delayed peaks compared with intravenous administration.

**FIG 3:**
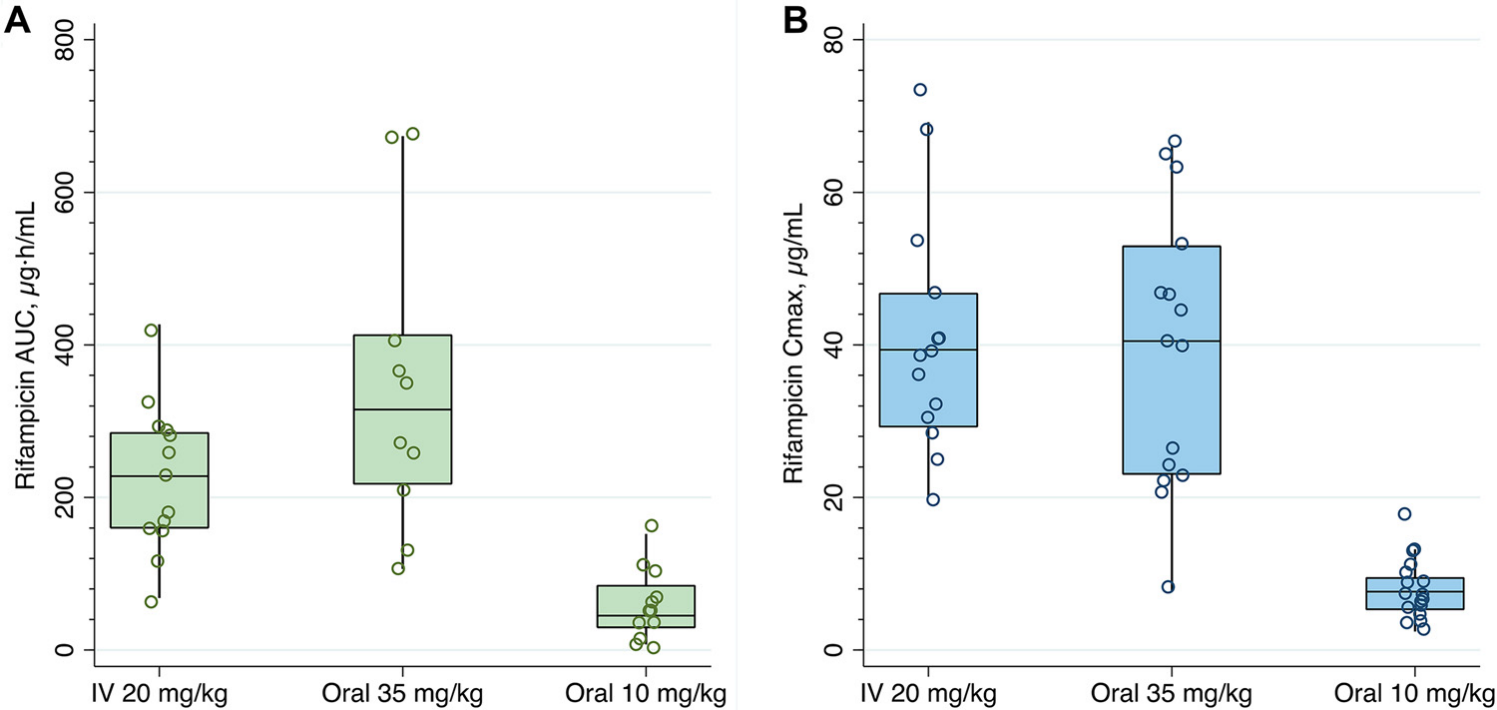
Comparison of exposures across dosing groups. Open circles are individual values for the AUC_0–24_ (A) and *C*_max_ (B), boxes indicate medians and interquartile ranges, and whiskers indicate the upper adjacent values (1.5× IQR).

**FIG 4:**
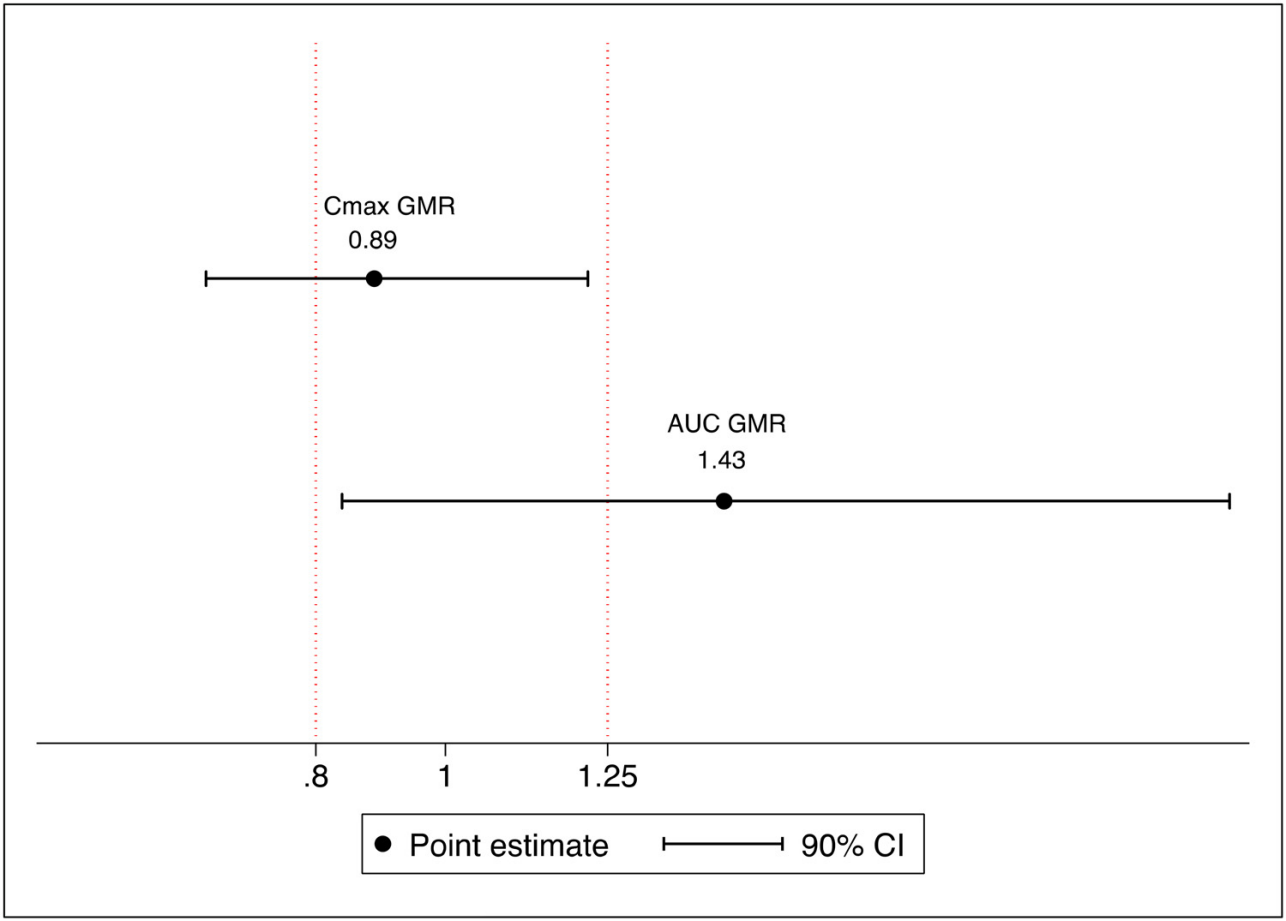
Bioequivalence plot. Point estimates of geometric mean ratios (GMRs) for the AUC_0–24_ and *C*_max_, with 90% confidence intervals, are shown, with vertical lines indicating bioequivalence margins. The reference measure is intravenous administration (û_oral_/û_i.v._); therefore, a value of >1 favors oral dosing.

Table 2 summarizes the estimated PK parameters from observed rifampicin concentrations, by dosing groups. The geometric mean AUC_0–24_ was 6.8-fold higher for the high-dose than for the standard-dose rifampicin group (*P* < 0.001) but was not significantly different between high-dose oral and i.v. administration (*P* = 0.22). The lowest AUC_0–24_ in the high-dose oral group (106.4 μg · h/ml) was 2.5-fold higher than the geometric mean AUC_0–24_ in the standard-dose group (42.9 μg · h/ml). The geometric mean *C*_max_ was 4.8-fold higher for the high-dose oral rifampicin group than for the standard-dose rifampicin group (*P* < 0.001) but similar between the high-dose oral and i.v. groups (*P* = 0.28). A comparison of exposures across dosing groups is shown in Fig. 3. The rifampicin AUC_0–24_ geometric mean ratio (GMR) was 1.44 (90% confidence interval [CI], 0.84 to 2.21) and the *C*_max_ GMR was 0.89 (90% CI, 0.63 to 1.23) for the high-dose oral group with respect to intravenous dosing (Fig. 4).

**TABLE 2:** Summary of PK parameters^*a*^

Parameter	Value for group			*P* value
	Standard dose, oral (*n* = 17)	High dose, oral (35 mg/kg) (*n* = 15)	i.v. (20 mg/kg) (*n* = 14)	
AUC_0–24_ (μg · h/ml)^*b*^				<0.001^*c*^
Geometric mean	42.9^*e*^	295.2	206.5	
95% CI	24.5–75.0	189.9–458.8	154.6–275.8	
Range	7.4–152.1	106.4–673.7	68.5–426.7	
Ratio to standard dose		6.9	4.8	

*C*_max_ (μg/ml)				<0.001^*c*^
Geometric mean	6.9^*e*^	34.7	38.6	
95% CI	5.2–9.2	25.2–47.8	31.2–47.6	
Range	2.4–18.1	7.7–66.0	20.2–74.0	
Ratio to standard dose		5.0	5.6	

Median *T*_max_ (h) (range)	2 (1–6)	3 (2–8)	1 (0.5–2)^*e*^	<0.001^*d*^

Median half-life (h) (range)	3.2 (2.6–13.3)	4.9 (2.1–21.6)^*e*^	2.6 (2.2–5.4)	0.01^*c*^

CL (liters/h)^*b*^				0.008^*c*^
Geometric mean	14.0^*e*^	7.4	6.6	
95% CI	8.1–24.3	4.6–11.8	4.9–8.6	
Range	4.9–100.7	2.2–21.4	3.9–17.5	
%CV	124.8	66.8	52.4	

*V* (liters)				0.01^*c*^
Geometric mean	72.9	55.2	27.8	
95% CI	37.2–142.9^*e*^	26.3–116.8	20.1–38.3	
Range	23.6–191.8	21.2–116.7	13–84.3	
%CV	184.2	150.9	59.8	

a CI, confidence interval; %CV, percent coefficient of variation; *V*, volume of distribution.

b Missing from 11 participants with unsuccessful intensive PK sampling and for whom there were not at least two observations in the elimination phase (standard dose, *n* = 5; high oral dose, *n* = 5; intravenous, *n* = 1).

c ANOVA after log transformation, with linear regression for pairwise comparisons.

d Kruskal-Wallis test.

e Comparator.

**FIG 5:**
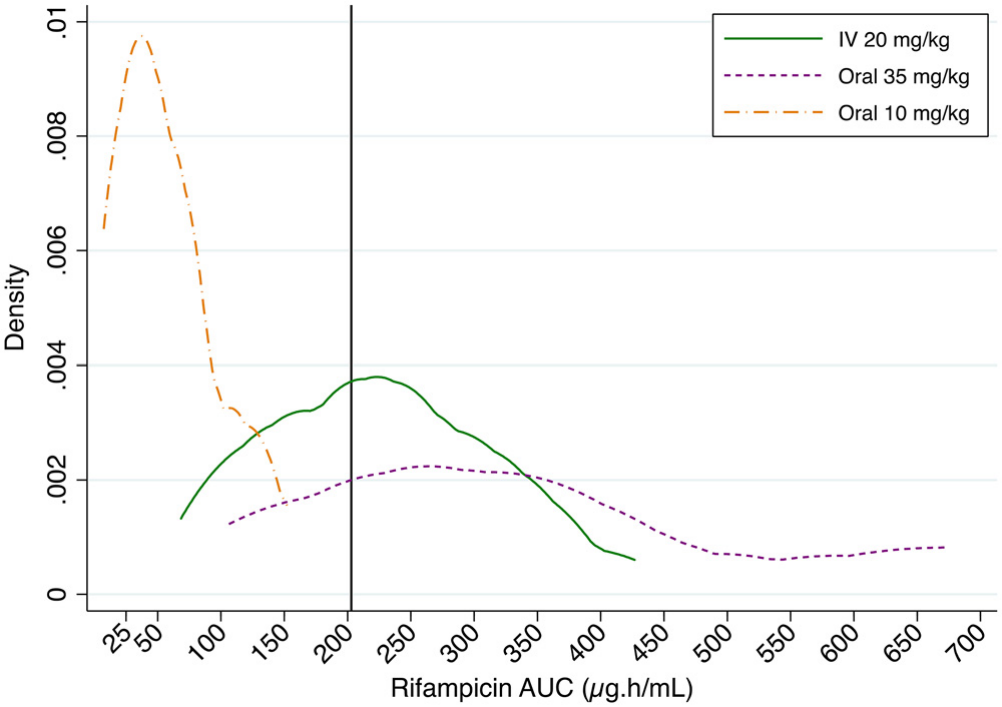
Probability density distributions for efficacy target attainment of rifampicin with different dosing strategies. The solid vertical line on the *x* axis represents the putative efficacy target AUC_0–24_ of 203 μg · h/ml.

The probabilities of efficacy target attainment, defined as an AUC_0–24_ of 203 μg · h/ml, were 80% (95% CI, 44 to 97%) for high-dose oral rifampicin and 54% (95% CI, 25 to 81%) for i.v. administration; none of the participants in the standard-dose arm achieved this target (Fig. 5).

**FIG 6:**
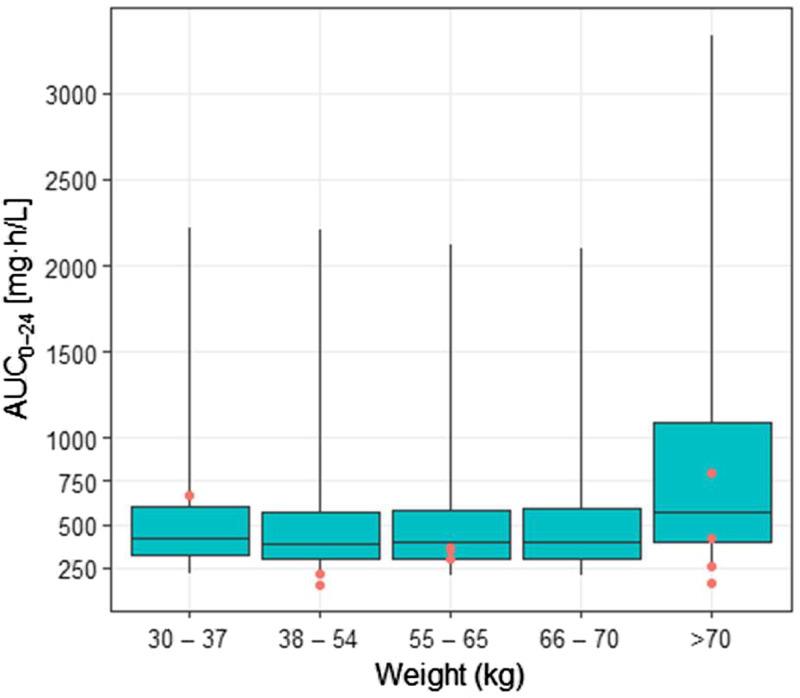
Simulated exposures across LASER-TBM weight bands for 35-mg/kg dosing, with observed exposures superimposed. Boxes indicate medians and interquartile ranges, and whiskers indicate ranges for simulated exposures derived from external cohorts, as described in the text. Red circles indicate observed exposures from the LASER-TBM cohort.

Exposures, measured by the AUC_0–24_, were not significantly different across weight bands for the high oral dose (*P* = 0.44), although this had poor precision because the number of participants in each band was small (Fig. 6). In an exploratory analysis, exposures were similar after the administration of crushed rifampicin via syringe for both the high-dose (geometric mean AUC_0–24_, 383.0 μg · h/ml [*n* = 2]) and standard-dose (geometric mean AUC_0–24_, 38.9 μg · h/ml [*n* = 4]) groups compared with those of participants who swallowed whole tablets (see Fig. S3 in the supplemental material).

## DISCUSSION

In our randomized controlled trial of South African adults with HIV-associated TBM, the plasma rifampicin AUC_0–24_ was higher after an oral 35-mg/kg dose than with intravenous administration of a 20-mg/kg dose over the first few days of TB treatment. Consistent with previous studies on both TBM (22) and pulmonary TB (11, 12, 20), there was a nonlinear dose-exposure relationship, with higher oral doses achieving supraproportional increases in exposures compared with standard oral dosing at 10 mg/kg.

The PK efficacy target for rifampicin in TBM is unknown, but it is plausible that dose optimization may lead to improved outcomes. Two small trials conducted in Indonesia suggested a survival benefit with the use of higher oral rifampicin doses of up to 30 mg/kg (equivalent to 1,350 mg in that population) and a significant and large effect with the use of intravenous dosing at 13 mg/kg (600 mg) (16, 22). A model-based meta-analysis of those data showed that rifampicin at 20 mg/kg given orally resulted in exposures similar to those with 13 mg/kg given intravenously and that this translated into a similar effect on TBM survival (18). That same analysis demonstrated an exposure-response relationship, and that effect was driven by the plasma AUC, similar to the microbiological response in phase 2b pulmonary TB studies (11, 12). Taken together, these findings suggest that outcomes in TBM can be improved with the use of higher rifampicin doses and that this is related to overall exposure, irrespective of the route of administration. Most participants randomized to high-dose oral rifampicin in our trial exceeded the putative efficacy target for TBM mortality (AUC of 203 μg · h/ml [18]); much fewer achieved this target in the intravenous group, and none did in the standard-dose group. This finding provides an additional rationale for evaluation of the oral 35-mg/kg dose in clinical efficacy trials.

The geometric mean AUC_0–24_ and *C*_max_ in the high-dose oral and intravenous groups in our study were similar to those reported in other populations (11, 20). Notably, our findings are consistent with a recent Ugandan trial that evaluated identical rifampicin dosing strategies in a predominantly HIV-positive cohort of TBM patients (*n* = 61). In that study, the geometric mean plasma rifampicin AUC_0–24_ values were 327 mg · h/liter with oral 35-mg/kg dosing and 217 mg · h/liter with intravenous 20-mg/kg dosing (23).

Rifampicin exposures predictably decline at steady state due to autoinduction and enhanced clearance (CL) with repeated dosing (20). Our study was designed to characterize rifampicin PK during the early phase of treatment with the assumption that optimizing exposures would be most critical for an antimycobacterial effect in this period. Although PK sampling occurred within the first 3 days of enrollment, the median time on rifampicin was 5 days at the time of the PK visit, when substantial autoinduction is expected to have occurred (19). Oral 35-mg/kg dosing would achieve even higher exposures at the start of therapy. In our informal bioequivalence analysis, the geometric mean AUC was ∼40% higher with oral 35-mg/kg than with intravenous 20-mg/kg administration, which could be explained by saturation of a first-pass effect at higher oral doses that would not apply to intravenous administration, resulting in a larger reduction in clearance and the resultant nonlinear dose-exposure relationship with oral dosing, particularly early in therapy. The higher clearance observed in the standard oral dose group supports this, as there is a much lower AUC relative to dose (CL is proportional to dose/AUC). As expected, the time to the maximal concentration (*T*_max_) was shorter with intravenous administration, but the *C*_max_ was similar to that with oral dosing at 35 mg/kg. An association between the plasma rifampicin *C*_max_ and survival was found in a small Indonesian TBM study (24) but was not reproduced in a larger Vietnamese trial (25) or in a pooled model-based analysis (18). A more rapid intravenous infusion could result in a higher *C*_max_ (26), but the safety and efficacy of this are not established and do not currently justify risks associated with venous catheterization.

We found large interindividual variability in rifampicin exposure, most pronounced in the oral dosing groups. This is a feature of rifampicin PK and relates to the effect of absorption delays on bioavailability and saturable kinetics (19, 20, 27). Although the AUC was on average significantly higher with 35-mg/kg oral dosing than with the standard dose, certain patients may not attain optimal exposures even at these higher doses. It was somewhat reassuring that, in our study population, the lowest rifampicin exposure in the 35-mg/kg group still exceeded the geometric mean AUC (and equaled the highest AUC) of the standard-dose group, suggesting a potential benefit from higher-dose rifampicin even in the context of highly variable bioavailability. Weight is an important source of rifampicin PK variability; patients with lower weights have relatively lower exposures for a given dose due to allometric scaling on clearance (28). We attempted to compensate for this by implementing a dosing strategy based on simulations using characteristics of a similar population that predicted equitable exposures for the high-dose oral group across modified weight bands. Notwithstanding the low number of participants receiving high-dose oral rifampicin in each weight band, exploratory analysis suggested no significant difference in observed exposures, providing partial validation of this approach. Another potential source of PK variability is the administration of crushed rifampicin tablets, which may affect dissolution characteristics and absorption (27). This is relevant in TBM, where patients frequently have reduced levels of consciousness. Reassuringly, the small group of participants (*n* = 6) who received crushed rifampicin in our study achieved exposures similar to those of participants swallowing whole tablets in their respective dosing groups; this is corroborated by findings from an Indonesian TBM cohort where 60% of participants were administered rifampicin via a nasogastric tube but achieved the expected increases in exposure at higher doses (22).

There are important limitations to consider when interpreting our findings. The sample size for evaluation of the primary outcome measure (AUC GMRs between high-dose oral and intravenous rifampicin, *n* = 29) was smaller than planned due to slow recruitment in the parent trial. However, in a *post hoc* power calculation using the original assumptions, this sample size would provide ∼80% power to detect a difference in the AUC of at least 30%, supporting the reliability of our main finding. It is unlikely that the direction of the effect would reverse to favor intravenous dosing, even with a larger sample size. The study was not powered to evaluate the impact of physiological or disease characteristics on PK variability; these analyses were not performed but are well known for rifampicin in similar populations. Rifampicin efficacy may depend on the protein-unbound fraction in TBM because only free drug crosses the blood-brain barrier. We measured total rifampicin concentrations, which was appropriate for our study given that we did not aim to evaluate the efficacy of dosing strategies. The free fraction of rifampicin is not expected to differ between oral and intravenous administration, even with large differences in exposure (29). We did not measure CSF rifampicin concentrations for this analysis because the primary objective was to compare plasma exposures of intravenous and oral rifampicin. Several studies have shown a correlation between plasma and CSF rifampicin exposures with oral dosing in TBM (16, 22, 25), and it is unlikely that CSF PK would be influenced by intravenous administration. Furthermore, plasma rifampicin exposure may be a better predictor of survival than CSF concentrations in TBM (18).

In summary, we have shown that in a population of African patients with HIV-associated TBM, the plasma rifampicin AUC_0–24_ was higher when dosed orally at 35 mg/kg than when dosed intravenously at 20 mg/kg, while the *C*_max_ values were similar. We also developed an empirical weight-based dosing strategy for high-dose oral rifampicin, which requires validation in a larger cohort. Our findings support high-dose oral rifampicin in future TBM trials.

## MATERIALS AND METHODS

### Parent trial and study population.

The parent study, called LASER-TBM, is a parallel-group, randomized, multiarm, open-label, phase 2a trial evaluating the safety of enhanced antimicrobial therapy with or without host-directed therapy for the treatment of HIV-associated TBM. Adults with confirmed HIV and newly diagnosed TBM (based on consensus definitions [30]) were recruited from four hospitals in Cape Town and Port Elizabeth, South Africa. Exclusion criteria included receipt of more than 5 days of antituberculosis medication, evidence of bacterial or cryptococcal meningitis, severe concurrent uncontrolled opportunistic disease, an estimated glomerular filtration rate (eGFR) of <20 ml/min (using the Cockcroft-Gault equation), an international normalized ratio (INR) of >1.4, clinical evidence of liver failure or decompensated cirrhosis, hemoglobin of <8.0 g/dl, <50 × 10^9^ platelets/liter, <0.5 × 10^9^ neutrophils/liter, and peripheral neuropathy of grade 3 or higher on the brief peripheral neuropathy score. Pregnancy was allowed if the gestational age was less than 17 weeks at enrollment.

Eligible and consenting participants were randomized at a ratio of 1.4:1:1 to either a standard-of-care control group or one of two experimental arms (relatively more participants were allocated to the control group as higher mortality was anticipated with the standard of care). Participants allocated to experimental arms 2 and 3 received additional rifampicin (total oral dose of 35 mg/kg/day) plus oral linezolid at 1,200 mg daily for the first 28 days, which was reduced to 600 mg daily for the next 28 days; those randomized to experimental arm 3 also received oral aspirin (1,000 mg daily). Study treatment was provided in all arms for 56 days, after which participants were referred back to public-sector facilities to complete standard therapy for HIV-associated TBM. All participants received antituberculosis chemotherapy as well as corticosteroids according to South African national TB management guidelines (36). The primary outcome for LASER-TBM was solicited adverse events and deaths in the experimental arms relative to the standard-of-care control arm at month 2; efficacy was a secondary outcome, determined at months 2 and 6.

### Design of the PK study.

A nested PK study was performed to compare plasma exposures (AUC and *C*_max_) of intravenous (i.v.) versus oral rifampicin. All consenting LASER-TBM participants allocated to experimental arms underwent a second randomization at the time of study entry, prior to receipt of the study drug, to receive either high-dose oral (35 mg/kg, according to the weight bands described below) or i.v. (20 mg/kg) rifampicin for the first 3 days of treatment. After day 3, all participants in the experimental arms continued high-dose oral rifampicin until day 56 (see Fig. S1 in the supplemental material).

Randomization was done in a 1:1 ratio using an electronic randomization tool and fully integrated with parent trial procedures. A parallel rather than a crossover design was chosen to remove the influence of rifampicin autoinduction on exposure over time, which increases rapidly over the first days of therapy (19). Due to the nature of the intervention and because the outcome measure is an objective PK endpoint, the allocation of intravenous versus oral rifampicin was unblinded.

Intensive plasma PK sampling took place during hospitalization on a single occasion within the first 3 days of enrollment. Serial venous blood samples were collected into K_3_EDTA Vacutainer tubes through a peripheral venous catheter predose and 0.5, 1, 2, 3, 6, 8 to 10, and 24 h after witnessed drug intake (or the start of i.v. infusion) and an overnight fast. Samples were centrifuged (1,500 × *g* for 10 min) within 1 h of collection. At least 1.5 ml of plasma was pipetted into polypropylene tubes and immediately frozen at −80°C. Sparse sampling was performed for participants who declined intensive sampling or in whom this failed. Plasma rifampicin concentrations were determined with a validated liquid chromatography-tandem mass spectrometry assay developed at the Division of Clinical Pharmacology, University of Cape Town. The assay was validated over the concentration range of 0.117 to 30.0 μg/ml. The combined accuracy and precision statistics of the limit of quantification for low-, medium-, and high-quality controls (three validation batches [*n* = 18]) were between 101% and 107% and between 2.7% and 3.7%, respectively.

Demographic and clinical data were collected from participants at the time of LASER-TBM study entry and at the PK visit. Data included biometrics, CD4 count, antiretroviral therapy (ART) status, TBM diagnosis (definite, possible, or probable by consensus definitions [30]), severity (grades 1 to 3 by the British Medical Research Council score), and functional status (modified Rankin score).

### Rifampicin dosing.

Oral rifampicin was provided as part of a fixed-dose combination (FDC) tablet with isoniazid, pyrazinamide, and ethambutol (Rifafour; Sandoz) according to standard WHO weight bands for the standard-dose group, with top-up of single-formulation tablets (Rimactane at 150 mg [Sandoz] and Eremfat at 600 mg [Riemser]) for the high-dose oral group. For participants unable to swallow whole tablets, the rifampicin was crushed, mixed with sterile water, and administered via a syringe. To account for the effect of allometry on clearance at lower weights, we performed simulations to determine the dose of rifampicin required to achieve the most equitable drug exposures across the weight range of 30 to 100 kg. Demographic data for a reference cohort of TB patients (*n* = 1,225), with or without HIV-1 coinfection, recruited in clinical studies conducted in West African countries and South Africa were used for the simulations (28, 31–33). An additional 12,250 virtual patients were generated using the weight and height distributions of the 1,225 patients to increase the number of patients with a weight close to the boundaries of the weight range. Parameter estimates of a population PK model for rifampicin were used to simulate (100 replicates) rifampicin exposures (20). Four dosing scenarios were evaluated using the weight-band-based dosing with 4-drug FDC tablets and extra rifampicin tablets, with each tablet containing 150 mg or 600 mg rifampicin. The FDC tablets were assumed to have 20% reduced bioavailability based on data from a clinical trial where the same formulation was used (34). The weight bands with the most balanced distribution in predicted exposures were used to dose oral rifampicin in the trial (Table S1 and Fig. S2). Intravenous rifampicin (Eremfat 600-mg vials; Riemser) was administered according to weight bands (Table S2) as a 1-h infusion, in accordance with instructions in the package insert, by nursing staff of the parent trial.

### Analysis.

The study was powered to detect a difference in exposure between oral and intravenous administration, defined as an AUC geometric mean ratio (GMR) of <0.8 (35). Assuming increased variability with oral dosing (percent coefficient of variance [%CV], 34) (20) versus intravenous dosing (%CV, 20), a sample size of 50 participants was planned to provide 80% power to demonstrate this with 90% two-sided confidence.

Demographic and clinical characteristics were summarized and compared using the Wilcoxon rank sum test for continuous variables and the χ^2^ test for dichotomous variables. Noncompartmental analysis was used to estimate rifampicin PK parameters from observed concentrations. The area under the concentration-time curve for the dosing interval (AUC_0–24_) was calculated using the trapezoidal method. The trough concentration (*C*_τ_) was defined as the plasma concentration 24 h after observed intake (actual or imputed, as described in the supplemental material). %CV was calculated as the mean/standard deviation × 100. Differences between log-transformed PK parameters across the three study groups were tested by one-way analysis of variance (ANOVA); the Kruskal-Wallis test was used for the time to the maximal concentration (*T*_max_). Linear regression was performed to compare pairwise coefficients between dosing groups. The means of log-transformed values for exposure parameters (log-normally distributed) were back-transformed to obtain geometric means; the GMR was calculated for the AUC_0–24_ and *C*_max_, with intravenous administration as the reference (û_oral_/û_i.v._). Fieller’s method was used to estimate 90% confidence intervals for the GMRs. We performed a *post hoc* PK/pharmacodynamic (PD) analysis for efficacy, on the suggestion of a reviewer. The probability of target attainment was calculated as the proportion of participants with PK exposures above a putative efficacy target of an AUC_0–24_ of 203 μg · h/ml (18). Probability distributions were constructed using kernel densities of observed AUC_0–24_ values, stratified by rifampicin dose. Statistical analysis was performed using Stata version 14.2 (StataCorp).

### Ethics.

This research was conducted in accordance with the Declaration of Helsinki and was approved by the University of Cape Town Human Research Ethics Committee (reference number 293/2018) and the Walter Sisulu University Human Research Committee (reference number 012/2019). The parent trial (LASER-TBM) is registered on ClinicalTrials.gov (identifier NCT03927313) and was approved by the South African Health Products Regulatory Authority (reference number 20180622).
