# Supplementary material for: Plasma pharmacokinetics of high dose oral versus intravenous rifampicin in patients with tuberculous meningitis: a randomized controlled trial
Source: Antimicrob Agents Chemother. Author manuscript; Available in PMC 2021 Jul 17. (PMC7611291; doi:10.1128/AAC.00140-21)
Supplement: Supplementary file 1 [file EMS124719-supplement-Supplementary_Material.pdf]

Plasma pharmacokinetics of high dose oral versus intravenous rifampicin in patients with tuberculous meningitis: a randomised controlled trial

SUPPLEMENTARY MATERIAL

Sean Wasserman[1,2#], Angharad Davis[1,3,4], Cari Stek[1,5,6], Maxwell Chirehwa[7], Stephani Botha[1], Remy Daroowala[1,5], Marise Bremer[1,8], Mpumi Maxebengula[1], Sonya Koekemoer[1], Rene Goliath[1], Amanda Jackson[1], Thomas Crede[6,9], Jonathan Naude[6,9], Patryk Szymanski[6,9], Yakoob Vallie[6,10], Muhammed S Moosa[6,10], Lubbe Wiesner[7], John Black[8], Graeme Meintjes[1,2], Gary Maartens[1,7], Robert J Wilkinson[1,2,3,4,5]

1. Wellcome Centre for Infectious Diseases Research in Africa, Institute for Infectious Disease and Molecular Medicine, University of Cape Town, Observatory, 7925, South Africa.
2. Division of Infectious Diseases and HIV Medicine, Department of Medicine, University of Cape Town, Observatory, 7925, South Africa.
3. Francis Crick Institute, Midland Road, London, NW11AT, UK.
4. Faculty of Life Sciences, University College London, Gower Street, London, WC1E 6BT, UK.
5. Department of Infectious Diseases, Imperial College, London, W12ONN, UK.
6. Department of Medicine, University of Cape Town, Cape Town, South Africa.
7. Division of Clinical Pharmacology, Department of Medicine, University of Cape Town, Observatory, 7925, South Africa.
8. Livingstone Hospital Complex, Eastern Cape Department of Health, Port Elizabeth, South Africa.
9. Mitchells Plain Hospital, Western Cape Department of Health, Cape Town, South Africa.
10. New Somerset Hospital, Western Cape Department of Health, Cape Town, South Africa.

IMPUTATION STRATEGY

Concentration-time profiles were inspected for each participant to compare pre-dose and 24-hour concentrations. In cases where the 24-hour concentration was missing, these were imputed as pre-dose concentrations if two prior observations were available in the elimination phase. The 24-hour concentration was considered highly unlikely to represent the true trough value where it exceeded the pre-dose concentration and was > 50% of the concentration at the prior sampling time point. This was based on the published elimination half-life of rifampicin,[16] and the assumption that the 24-hour concentration would therefore fall below the 6- or 8-hour concentration in the absence of additional dosing. In these cases, the 24-hour concentration was imputed from the pre-dose concentration. Where the pre-dose concentration exceeded the 24-hour concentration by > 2-fold, indicating late dosing prior to the PK visit, the pre-dose concentration was replaced by $C24*K_e$ to adjust for contribution to AUC. Concentrations reported as below the limit of assay quantification (BLQ) were imputed as 50% of the lower limit of detection (i.e. 0.585 μg/mL).

FIGURES

Figure S1. Trial schema

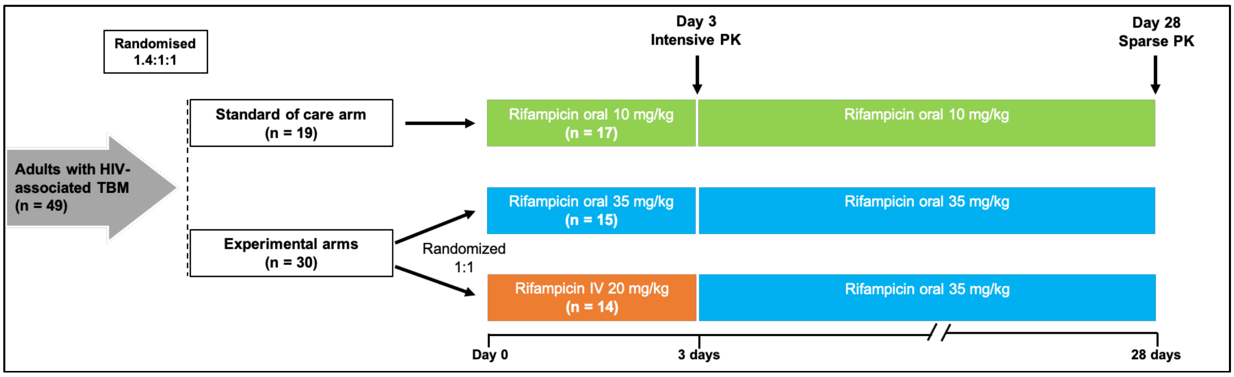

Figure S2. Simulations showing balanced exposures across weight bands with LASER-TBM dosing table

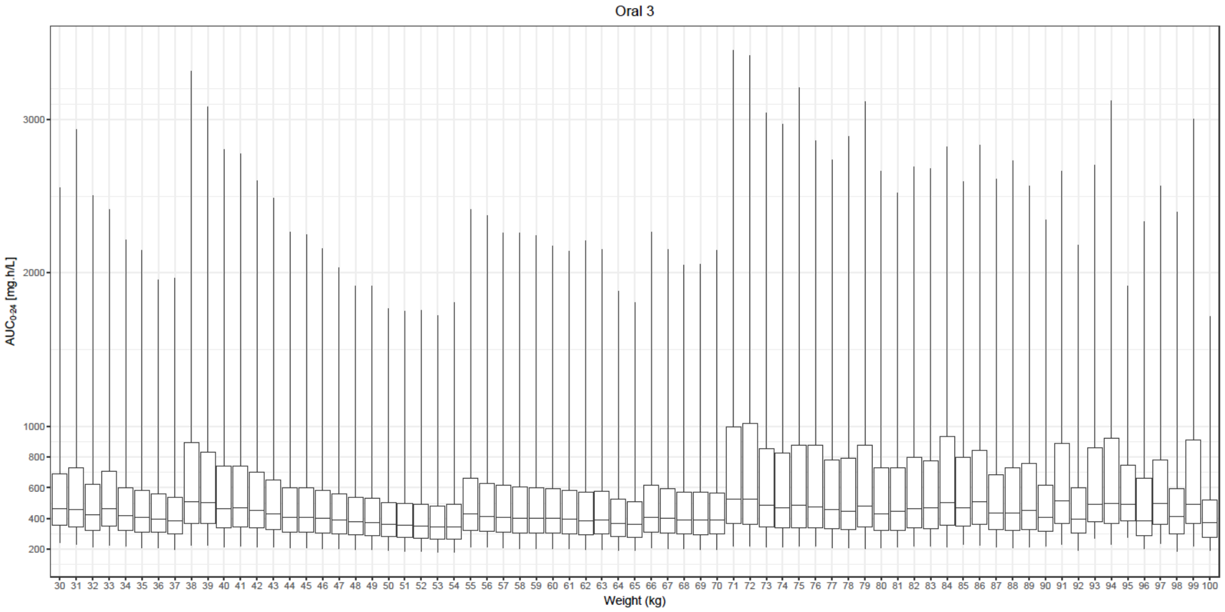

Figure S3. Exposures from swallowed versus crushed administration of rifampicin tablets for oral 35 mg/kg (Fig. S3A) and 10 mg/kg (Fig. S3B).

Fig S3A

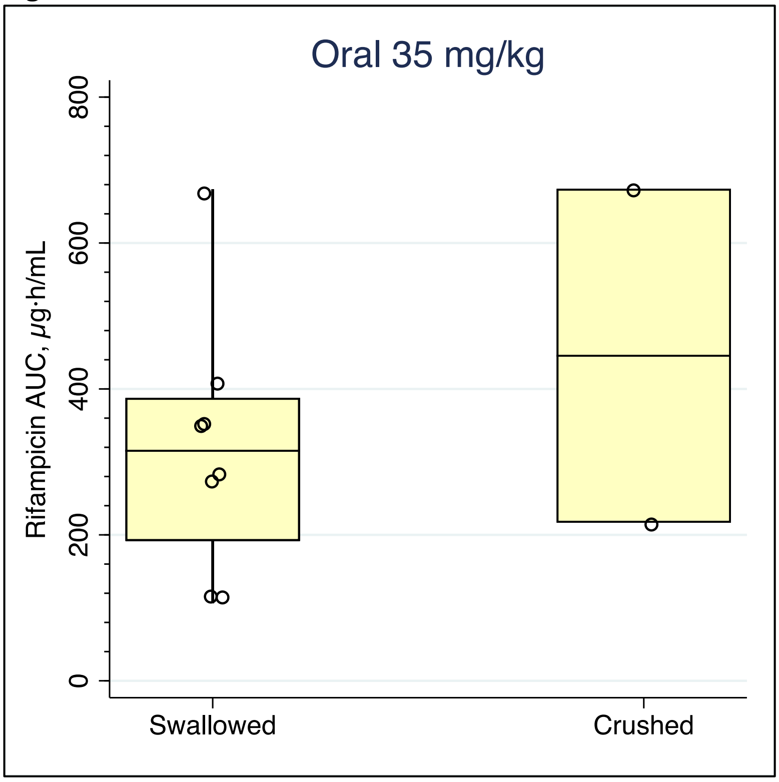

Fig S3B

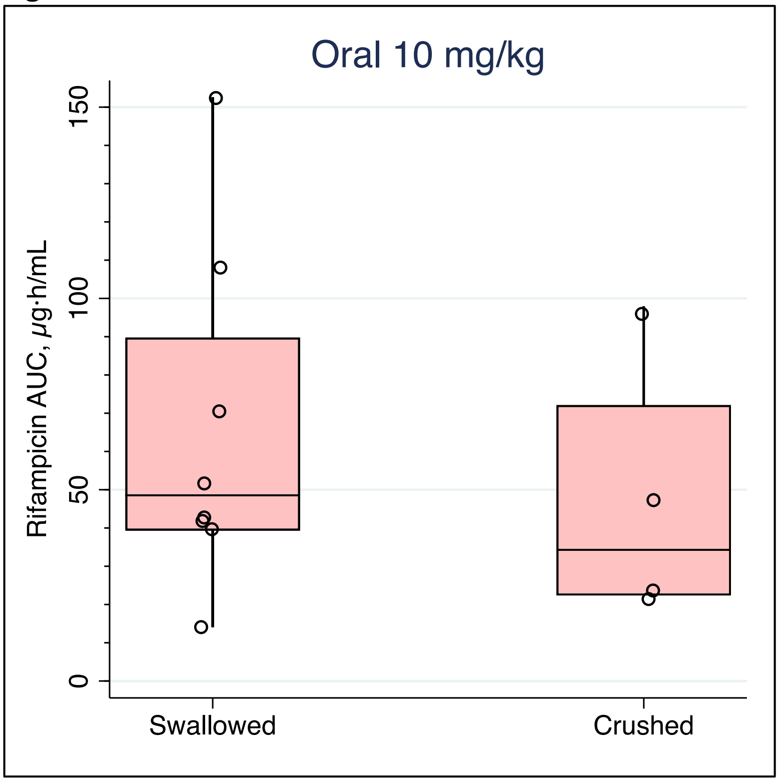
Oral 10 mg/kg
Rifampicin AUC, μg·h/mL
150
100
50
0
Swallowed
Crushed

TABLES

Table S1. Weight bands for oral rifampicin dosing

LASER-TBM bands	Band 1	Band 2	Band 3	Band 4	Band 5
**Weight range**	30 – 37 kg	38 – 54 kg	55- 65 kg	66 - 70	> 70 kg
**R_{10}HZE (WHO)**	300	450	600	600	750
**R_{25} additional**	1200	1350	1500	1650	1950
**Total RIF (~35 mg/kg)**	1500	1800	2100	2250	2700

Table S2. Weight bands for intravenous rifampicin dosing

	Band 1	Band 2	Band 3	Band 4	Band 5	Band 6
Weight range	30 – 33 kg	34 - 37 kg	38 – 54 kg	55- 65 kg	66 - 70 kg	> 70 kg
HZE tabs	2	2	3	4	4	5
R_{20} IV	900	1050	1200	1350	1500	1650
Total Rif	900	1050	1200	1350	1500	1650
